# P15 Bacteriological profile and antimicrobial resistance trends in chronic suppurative otitis media (CSOM) in India: comprehensive review (1961–2026)

**DOI:** 10.1093/jacamr/dlag102.021

**Published:** 2026-06-26

**Authors:** Saurabh Varshney, Pratima Gupta, Shyam Kishor Kumar, Aroop Mohanty, Mithilesh Kumar Jha, Minakshi Singh

**Affiliations:** All India Institute of Medical Sciences Rishikesh, India; All India Institute of Medical Sciences Rishikesh, India; All India Institute of Medical Sciences Deoghar, India; All India Institute of Medical Sciences Gorakhpur, India; All India Institute of Medical Sciences Deoghar, India; All India Institute of Medical Sciences Rishikesh, India

## Abstract

**Background:**

Chronic suppurative otitis media (CSOM), characterized by persistent middle ear suppuration through tympanic membrane perforation for ≥2 weeks, remains a major public health challenge in India with prevalence ranging from 1.4% to 14.7%, classifying India as a WHO high-prevalence zone. The earliest CSOM study from Uttar Pradesh in 1961 reported 14.7% prevalence among schoolchildren. This 65-year review analyses how CSOM pathogens and their resistance patterns have evolved across India's regions.

**Methods:**

Review studies from Karnataka, Bhopal, Agartala, Bhopal, Panipat, Rajasthan, West Bengal, and other centres revealed that early studies used manual culture and Kirby-Bauer disc diffusion (CLSI M100). Recent studies incorporated VITEK-2/MicroScan automated systems for identification and AST (6-12h), MALDI-TOF MS for rapid proteomic speciation (<30 min), and Etest strips for precise MIC determination of anaerobes. Data were analysed by geographic region, pathogen predominance, resistance evolution, demographic patterns, and diagnostic methodology.

**Results:**

Across Indian studies, *Pseudomonas aeruginosa* (19.9–67.5%) and *Staphylococcus aureus* (11.3–51.9%) consistently emerged as the predominant pathogens across all geographic regions and time periods, while anaerobic bacteria like *Bacteroides fragilis* and *Finegoldia magna*, along with fungi including *Aspergillus species* and *Candida species were identified*. Demographic analysis revealed male predominance with male-to-female ratios from 1.3:1 to 1.6:1, bilateral ear involvement in 22.5%, and peak disease among individuals aged 11-30 years. Antimicrobial resistance patterns showed dramatic escalation over time, with *P. aeruginosa* ciprofloxacin resistance increasing from 20% in the early 2000s to 78.9% in recent studies, alongside gentamicin resistance rates of 26–64%, although this pathogen maintained greater than 90% susceptibility to colistin, polymyxin B, carbapenems, and piperacillin/tazobactam. *S. aureus* exhibited methicillin-susceptible strains predominating over MRSA in 9 out of 10 studies, with vancomycin and linezolid retaining greater than 90% activity while demonstrating 78.9% ciprofloxacin resistance.

**Conclusions:**

Diagnostic methodology evolution has profoundly impacted CSOM microbiology interpretation. Early studies (1961–1990s) using conventional methods yielded broad genus-level reporting prone to misidentification (*Pseudomonas* spp. versus *Stenotrophomonas*); post-2010s VITEK-2/MicroScan/MALDI-TOF revolution revealed colistin MIC creep (0.5→2 mg/L), MRSA clonal types (ST22), biofilm persistence, enhanced fungal/anaerobe recovery which was previously missed, enabling 25–40% improved therapeutic outcomes through rapid de-escalation from empirical fluoroquinolone failures. Modern systems facilitate resistance gene correlation (blaVIM, NDM, mecA) guiding precision stewardship.

**Conclusions:**

The persistent predominance of *P. aeruginosa* and *S. aureus* over 65 years highlights enduring socioeconomic challenges driving CSOM in India. The escalating multidrug resistance crisis necessitates immediate development of region-specific antibiograms to guide empirical therapy—prioritizing colistin and carbapenem-based topical formulations for gram-negative pathogens alongside vancomycin or linezolid for *S. aureus*. The automated diagnostic revolution (MALDI-TOF MS/VITEK-2/MicroScan) has transformed CSOM precision. Future priorities include whole-genome sequencing for carbapenemase tracking, biofilm profiling, and pan-regional surveillance to combat hearing loss and resistance.

